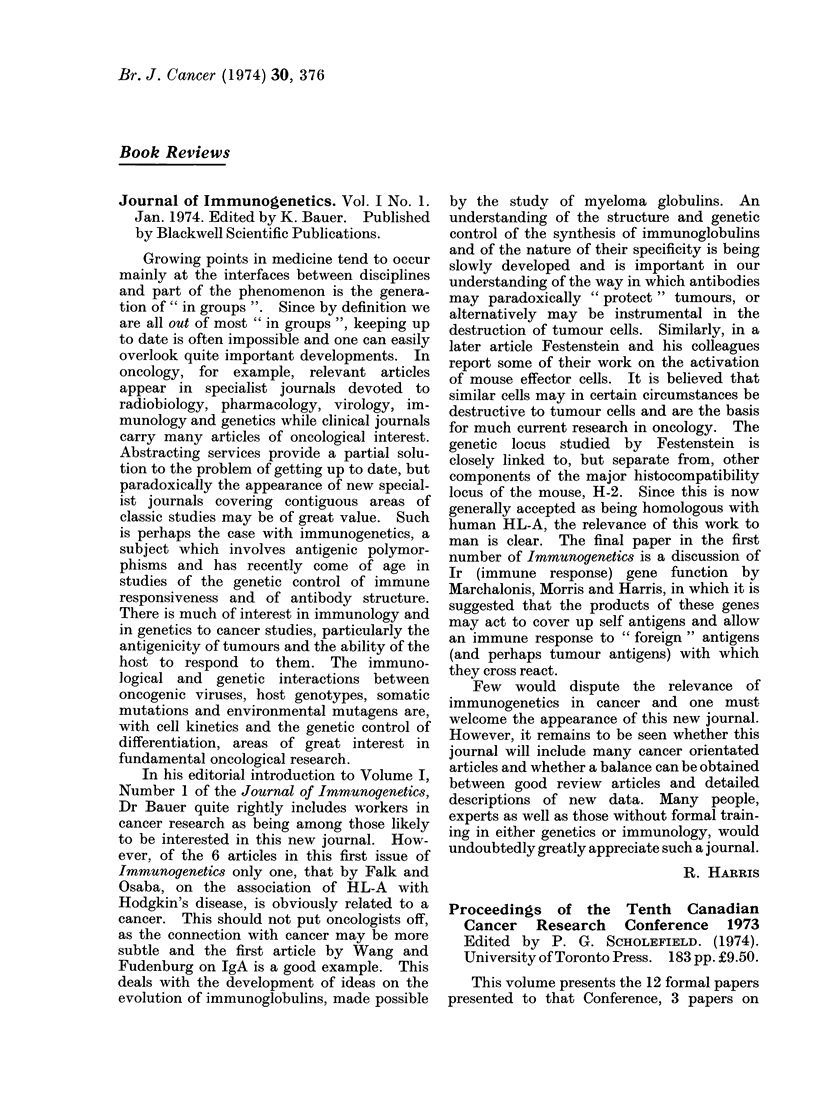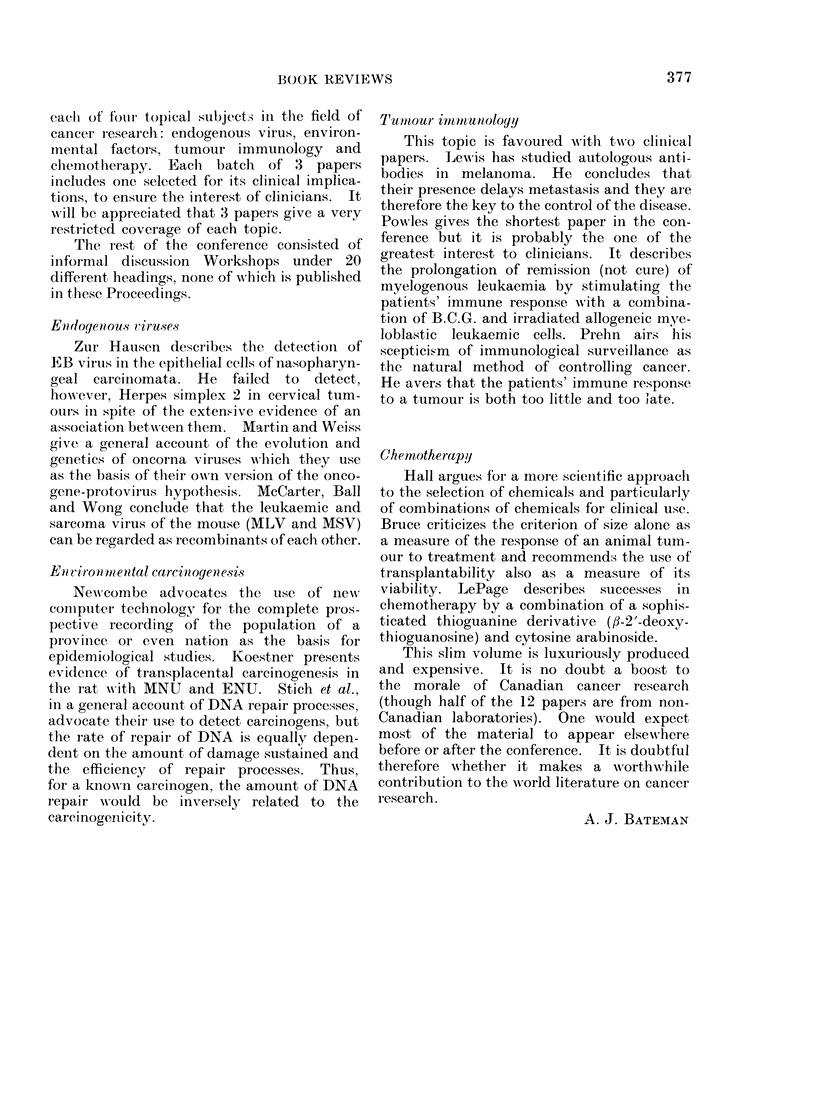# Proceedings of the Tenth Canadian Cancer Research Conference 1973

**Published:** 1974-10

**Authors:** A. J. Bateman


**Proceedings of the Tenth Canadian Cancer Research Conference 1973** Edited by P. G. Scholefield. (1974). University of Toronto Press. 183 pp. £9.50.

This volume presents the 12 formal papers presented to that Conference, 3 papers on each of four topical subjects in the field of cancer research: endogenous virus, environmental factors, tumour immunology and chemotherapy. Each batch of 3 papers includes one selected for its clinical implications, to ensure the interest of clinicians. It will be appreciated that 3 papers give a very restricted coverage of each topic.

The rest of the conference consisted of informal discussion Workshops under 20 different headings, none of which is published in these Proceedings.

*Endogenous viruses*

Zur Hausen describes the detection of EB virus in the epithelial cells of nasopharyngeal carcinomata. He failed to detect, however, Herpes simplex 2 in cervical tumours in spite of the extensive evidence of an association between them. Martin and Weiss give a general account of the evolution and genetics of oncorna viruses which they use as the basis of their own version of the oncogene-protovirus hypothesis. McCarter, Ball and Wong conclude that the leukaemic and sarcoma virus of the mouse (MLV and MSV) can be regarded as recombinants of each other.

*Environmental carcinogenesis*

Newcombe advocates the use of new computer technology for the complete prospective recording of the population of a province or even nation as the basis for epidemiological studies. Koestner presents evidence of transplacental carcinogenesis in the rat with MNU and ENU. Stich *et al.*, in a general account of DNA repair processes, advocate their use to detect carcinogens, but the rate of repair of DNA is equally dependent on the amount of damage sustained and the efficiency of repair processes. Thus, for a known carcinogen, the amount of DNA repair would be inversely related to the carcinogenicity.

*Tumour immunology*

This topic is favoured with two clinical papers. Lewis has studied autologous antibodies in melanoma. He concludes that their presence delays metastasis and they are therefore the key to the control of the disease. Powles gives the shortest paper in the conference but it is probably the one of the greatest interest to clinicians. It describes the prolongation of remission (not cure) of myelogenous leukaemia by stimulating the patients' immune response with a combination of B.C.G. and irradiated allogeneic myeloblastic leukaemic cells. Prehn airs his scepticism of immunological surveillance as the natural method of controlling cancer. He avers that the patients' immune response to a tumour is both too little and too late.

*Chemotherapy*

Hall argues for a more scientific approach to the selection of chemicals and particularly of combinations of chemicals for clinical use. Bruce criticizes the criterion of size alone as a measure of the response of an animal tumour to treatment and recommends the use of transplantability also as a measure of its viability. LePage describes successes in chemotherapy by a combination of a sophisticated thioguanine derivative ($\beta$-2′-deoxythioguanosine) and cytosine arabinoside.

This slim volume is luxuriously produced and expensive. It is no doubt a boost to the morale of Canadian cancer research (though half of the 12 papers are from non-Canadian laboratories). One would expect most of the material to appear elsewhere before or after the conference. It is doubtful therefore whether it makes a worthwhile contribution to the world literature on cancer research.

A. J. BATEMAN